# Breaking bad aggregates: How a DNA aptamer cleans up Parkinson’s disease

**DOI:** 10.1016/j.omtn.2024.102296

**Published:** 2024-08-29

**Authors:** Maxim V. Berezovski

**Affiliations:** 1 Department of Chemistry and Biomolecular Sciences, University of Ottawa, 10 Marie-Curie, Ottawa, ON K1N 6N5, Canada

## Main text

The aggregation and misfolding of α-synuclein (αSyn) protein represent a neuropathological hallmark of Parkinson’s disease (PD). This study by Maria DeRosa and her team from Carleton University in Canada introduces a novel selection method for identifying DNA aptamers that exhibit a high affinity for monomeric αSyn and effectively inhibit its aggregation.^1^ Among these, the aptamer a-syn-1 demonstrated significant potential in both *in vitro* and *in vivo* models, offering a promising strategy for PD treatment.

PD is a progressive neurodegenerative disorder affecting millions worldwide, primarily the elderly.^2^ Characterized by motor symptoms such as tremors, rigidity, and bradykinesia, PD stems from the loss of dopaminergic neurons in the substantia nigra. A key pathological feature of PD is the misfolding and aggregation of αSyn, leading to the formation of insoluble fibrils and neuronal death.^3^ Traditional therapies focus on symptom management rather than halting disease progression.^4^ Hence, there is a pressing need for innovative approaches that target the underlying mechanisms of PD.

The authors employed a modified version of systematic evolution of ligands by exponential enrichment (SELEX) approach to identify DNA aptamers capable of binding to monomeric αSyn and preventing its aggregation. The selection process involved incubating a DNA library with αSyn, encouraging aggregation, and isolating aptamers that retained αSyn in its monomeric state. Among the identified aptamers, a-syn-1 stood out for its ability to inhibit αSyn aggregation *in vitro*, confirmed through transmission electron microscopy (TEM) and Thioflavin T fluorescence assays.

In cellular models using SH-SY5Y cells, a-syn-1 significantly reduced intracellular αSyn aggregation compared to control groups. Furthermore, systemic delivery of a-syn-1 via a liposome vehicle in transgenic mice overexpressing the human A53T variant of αSyn resulted in decreased levels of aggregated αSyn in key brain regions, including the prefrontal cortex, caudate, and substantia nigra. Interestingly, the authors used a second aptamer that recognizes the transferrin receptor, which is highly expressed on the blood-brain barrier (BBB) and delivers liposomes to the brain. These findings underscore the potential of a-syn-1 as a therapeutic agent capable of crossing the BBB and targeting αSyn pathology *in vivo*.

The implications of this study for the field of PD research and treatment are profound. The ability to inhibit αSyn aggregation not only addresses a fundamental aspect of PD pathology but also opens new avenues for the development of targeted therapies. However, several important questions remain. The long-term effects and potential toxicity of repeated aptamer administration need thorough investigation. Additionally, understanding the precise mechanisms by which a-syn-1 disrupts αSyn aggregation could enhance the design of more effective therapeutic agents.

The introduction of aptamers as therapeutic agents in neurodegenerative diseases marks a significant advancement. Aptamers offer several advantages, including high specificity, ease of synthesis, and the potential for chemical modifications. In the context of PD, aptamers like a-syn-1 could complement existing treatments, providing a multifaceted approach to managing the disease. Moreover, the use of aptamers extends beyond treatment to diagnostic applications, offering the potential for early detection of αSyn aggregation and timely intervention.

In conclusion, the study by McConnell et al. presents a groundbreaking approach to inhibiting αSyn aggregation, with significant implications for PD treatment. The identification of a-syn-1 as a potent inhibitor of αSyn aggregation in both cellular and animal models represents a critical step toward developing effective therapeutic strategies. Future research should focus on optimizing the delivery and efficacy of aptamers, exploring their long-term effects, and integrating them into comprehensive treatment regimens for PD. This work not only enhances our understanding of PD pathology but also paves the way for innovative therapies that could alter the course of neurodegenerative diseases.

## Declaration of interests

The authors declare no competing interests.
